# Life Satisfaction and Morbidity among Postmenopausal Women

**DOI:** 10.1371/journal.pone.0147521

**Published:** 2016-01-22

**Authors:** Pyry S. Lukkala, Risto J. Honkanen, Päivi H. Rauma, Lana J. Williams, Shae E. Quirk, Heikki Kröger, Heli Koivumaa-Honkanen

**Affiliations:** 1 School of Medicine, Faculty of Health Sciences, University of Eastern Finland (UEF), Kuopio, Finland; 2 Bone and Cartilage Research Unit, Surgery, Institute of Clinical Medicine, UEF, Kuopio, Finland; 3 Social Pharmacy, School of Pharmacy, Faculty of Health Sciences, UEF, Kuopio, Finland; 4 IMPACT Strategic Research Centre, School of Medicine, Deakin University, Geelong, Australia; 5 Department of Orthopaedics, Traumatology and Handsurgery, Kuopio University Hospital (KUH), Kuopio, Finland; 6 Institute of Clinical Medicine, Psychiatry, UEF, Kuopio, Finland; 7 Department of Psychiatry: KUH, Kuopio, South-Savonia Hospital District, Mikkeli, North Karelia Central Hospital, Joensuu, SOSTERI, Savonlinna, SOTE, Iisalmi, Lapland Hospital District, Rovaniemi, Finland; University of Rennes-1, FRANCE

## Abstract

**Objective:**

To investigate associations between morbidity and global life satisfaction in postmenopausal women taking into account type and number of diseases.

**Materials and Methods:**

A total of 11,084 women (age range 57–66 years) from a population-based cohort of Finnish women (OSTPRE Study) responded to a postal enquiry in 1999. Life satisfaction was measured with a 4-item scale. Self-reported diseases diagnosed by a physician and categorized according to ICD-10 main classes were used as a measure of morbidity. Enquiry data on health and lifestyle were used as covariates in the multivariate logistic models.

**Results:**

Morbidity was strongly associated with life dissatisfaction. Every additional disease increased the risk of life dissatisfaction by 21.1% (p < .001). The risk of dissatisfaction was strongest among women with mental disorders (OR = 5.26; 95%CI 3.84–7.20) and neurological disorders (OR = 3.62; 95%CI 2.60–5.02) compared to the healthy (each p < .001). Smoking, physical inactivity and marital status were also associated with life dissatisfaction (each p < .001) but their introduction to the multivariate model did not attenuate the pattern of associations.

**Conclusions:**

Morbidity and life dissatisfaction have a disease-specific and dose-dependent relationship. Even if women with mental and neurological disorders have the highest risk for life dissatisfaction, monitoring life satisfaction among aging women regardless of disorders should be undertaken in order to intervene the joint adverse effects of poor health and poor well-being.

## Introduction

The interplay between psychological factors and physical health was anticipated in the World Health Organization (WHO) definition of health (“…state of complete physical, mental and social well-being and not merely the absence of disease”) [1]. Later, self-rated health was proposed to include components of physical and psychological well-being [2] and to be a strong predictor of mortality [3,4]. Since, the relationship between psychological factors and somatic health has been a growing field of research interest [5–8].

Life satisfaction is a subjective, cognitive evaluation of an individual’s life as a whole [9]. Healthy individuals appear to be more satisfied and happier than those who are physically unwell [10–13]. Life satisfaction and happiness are indicators of subjective well-being (SWB), which is one of the main dimensions of mental health [14]. A brief 4-item life satisfaction scale (LS) has proven to be broadly linked with indicators of mental health, particularly with depression [15–17] including both sub-threshold depressive symptoms, but also the positive pole of mental health. Most importantly, LS has identified those with increased risk for several adverse health outcomes such as mortality and suicide as well as both psychiatric and somatic disability and morbidity [18–22].

It is plausible that the effects of diseases on SWB differ by the type and number of diseases; yet few studies discuss and compare these differences. Previous research has mainly focused on specific symptoms, health disorders or subpopulations [10,11,23–27]. Furthermore, morbidity data has commonly been classified and aggregated into broad categories of psychiatric and somatic disorders. Thus, a more detailed comparative inspection of disease groups in respect to SWB is needed.

In the present study we investigated whether and how LS and a broad range of disease categories and multimorbidity are associated in women approaching older age. As medical conditions tend to cluster with age [28] and postmenopausal depression is common [29,30], further research is needed. Our hypothesis was that significant associations will be found between types of morbidity and life satisfaction. Knowledge of these disease-specific relationships is useful and needed for health promotion, disease prevention and prevention of premature death.

## Materials and Methods

### 2.1 Study design

The study population originated from the Kuopio Osteoporosis Risk Factor and Prevention (OSTPRE) Study cohort with a target population of all the 14,220 women born in 1932–41 and resident in the Province of Kuopio at baseline in 1989. A total of 13,100 (92%) women responded to the baseline enquiry. At the 10-year follow-up conducted in 1999, 11,537 women responded to the enquiry. Included in the current cross-sectional study are the 11,084 women who responded and had complete data on all the four items of the LS scale in 1999.

The OSTPRE study was reviewed and approved by the Ethics Committee of the Hospital District of North Savo and Kuopio University Hospital in 1986, 1994, 1997, 2002, 2008 and 2009. Permission for national registers was approved in 2010 and 2012 (THL). All regulations and measures of confidentiality and data security are strictly enforced according to university guidelines, national legislation and in accordance with the Declaration of Helsinki. All study subjects have given informed written consent and have been assigned a code number ensuring anonymity. All study data is stored at the study centre and secured in accordance with the high standard of UEF data procedures.

### 2.2 Life satisfaction scale

Life satisfaction was assessed using the 4-item scale (range 4–20, higher score indicating lower life satisfaction). The items include current feelings of 1) interest and 2) happiness in life, 3) ease of living; and 4) feelings of loneliness. The response categories have been presented in detail elsewhere [15]. LS was used as a continuous, a 3-category variable (the satisfied, LS = 4–6; the intermediate group, LS = 7–11; the dissatisfied, LS = 12–20) and a 2-category variable (the satisfied, LS 4–11; the dissatisfied, LS = 12–20) [15].

Previously, the response rate of the 4-item life satisfaction scale has been around 96–98% in a large general population cohort [15,19,20]. In the present study, it was 96%. The LS-scale has proved its validity as a measure of both treatment outcome [31] and a global SWB in several studies. It has been shown to be correlated strongly with depression [15]. Its internal consistency is considered good [32] and it has also been shown to be associated with Health Related Quality of Life (HRQL) and sense of coherence (SOC) both in cross-sectional and longitudinal settings [33–35].

### 2.3 Chronic diseases and other variables

Information regarding chronic diseases was obtained at the 10-year OSTPRE follow-up enquiry via the following question: “Have you (had) any of the following diseases diagnosed by a physician?”. Thirty-four chronic diseases were listed followed by a blank space for additional diseases. A total of 96 different diseases were reported by the participants. The number of reported chronic diseases was used as the measure of (multi)morbidity and categorized as follows: none, 1–2, 3–4 and ≥5 diseases. Diseases were classified and grouped according to International Classification of Diseases, ICD-10. Small ICD main classes were combined into a category *Other diseases*.

Lifestyle and socio-demographic variables were also obtained at the 10-year enquiry. Leisure time physical activity was considered as a dichotomous (no/yes) variable. Use of alcohol as absolute alcohol was calculated and categorized as none/≤360/>360 grams per month. Smoking was categorized as none/≤10/>10 cigarettes per day. Body Mass Index (BMI) was used as a continuous variable. Marital status was considered as a dichotomous variable (married/domestic partnership vs. single/divorced/widowed).

### 2.4 Statistical analyses

Statistical analyses were performed using the SPSS statistical package 21.0 for Windows (SPSS Inc., Chicago, IL, USA). Differences between means of continuous variables were examined with ANOVA and proportions of categorical variables with the Chi-squared test (Table 1).

**Table 1:** Characteristics of the study population by the 3-category life satisfaction (LS).

	All	Satisfied	Intermediate	Dissatisfied
	LS 4–20	LS 4–6	LS 7–11	LS 12–20
*Means (SD) of continuous variables*	(N = 11,084)	(N = 2,685)	(N = 6,960)	(N = 1,439)
Age, years	62.3 (2.9)	62.1 (2.8)	62.3 (2.9)*	62.2 (3.0)
Height, cm	160.3 (5.4)	160.8 (5.3)	160.1 (5.4)***	160.2 (5.6)**
Weight, kg	71.0 (12.4)	70.2 (11.6)	71.0 (12.2)*	72.8 (14.4)***
BMI, kg/m^2^	27.6 (4.6)	27.2 (4.3)	27.6 (4.5)***	28.3 (5.5)***
Number of health disorders	2.4 (1.8)	2.0 (1.7)	2.4 (1.8)***	3.0 (2.0)***
*Proportions (%) of category variables*
Smoking, %	8.2 ●	6.1	8.1	12.2
Alcohol use, %	52.2 ●	57.1	51.8	44.4
Leisure time physical activity, %	70.0 ●	77.4	69.4	58.6
Poor self-rated health, %	7.8 ●	2.0	6.4	25.4
≥5 health disorders, %	12.1 ●	8.0	11.9	20.3

Means ANOVA (Post Hoc, Tukey HSD) vs. the satisfied

* p < .05

** p < .01

*** p < .001.

Proportions Chi-Square

● p < .001.

Binary logistic regression was used to assess associations between morbidity and LS. In Table 2, the relationship between multimorbidity and LS was examined using the 3-category LS (LS 7–11 vs. LS 4–6 and LS 12–20 vs. LS 4–6) as the outcome and 4-category number of diseases as the exposure. In Table 3, with the disease class as the exposure variable, 2-category LS (LS 12–20 vs. LS 4–11) was used as the outcome (Table 3). For the fully adjusted model, the covariates and possible confounders were chosen based on previous literature and/or whether each variable of interest was significantly associated with LS and health status in the unadjusted models.

**Table 2:** Risk (OR) of belonging to the intermediate (LS 7–11) or to the dissatisfied (LS 12–20) group by number of diseases with the healthy as a reference group.

	Intermediate OR [95%CI]		Dissatisfied OR [95%CI]	
	(LS 7–11 vs. LS 4–6)		(LS 12–20 vs. LS 4–6)	
	Unadjusted	Adjusted^1^	Unadjusted	Adjusted^1^
No diseases (n = 1220)	1.00	1.00	1.00	1.00
1–2 diseases (n = 4589)	1.23 [1.08–1.41]	1.22 [1.06–1.41]	1.51 [1.20–1.91]	1.45 [1.12–1.88]
3–4 diseases (n = 2468)	1.61 [1.39–1.87]	1.59 [1.35–1.86]	2.91[2.28–3.71]	2.69 [2.04–3.53]
≥5 diseases (n = 1007)	1.99 [1.64–2.42]	1.96 [1.59–2.41]	5.19 [3.93–6.84]	4.53 [3.31–6.21]

^1^ Adjusted with continuous BMI, 3-category smoking and alcohol use, 2- category physical activity and marital status.

**Table 3:** Mean LS scores and risks (OR) of life dissatisfaction (LS 12–20 vs. LS 4–11) for each ICD-10 main class with the healthy as a reference group.

			Unadjusted model			Adjusted model		
	N	Mean LS^1^	OR	95% CI	p-value	OR_adj_^2^	95% CI	p-value
No diseases (reference)	1220	7,52	1.00			1.00		
Infectious diseases	159	8,18*	1.80	1.10–2.94	.019	1.86	1.10–3.14	.020
Neoplasms	825	8,33**	2.17	1.66–2.84	< .001	2.18	1.62–2.92	< .001
Endocrine and metabolism disorders	4590	8,22**	1.80	1.45–2.22	< .001	1.76	1.40–2.22	< .001
Mental and behavioural disorders	294	9,86**	5.26	3.84–7.20	< .001	4.26	2.94–6.15	< .001
Neurological disorders	299	9,18**	3.62	2.60–5.02	< .001	3.36	2.33–4.84	< .001
Eye diseases	294	8,93**	3.37	2.41–4.71	< .001	3.07	2.11–4.48	< .001
Cardiovascular diseases	4881	8,28**	1.89	1.53–2.33	< .001	1.71	1.36–2.16	< .001
Respiratory diseases	1231	8,42**	1.98	1.54–2.54	< .001	1.78	1.35–2.36	< .001
Digestive system disorders	509	8,70**	2.60	1.94–3.50	< .001	2.74	1.99–3.78	< .001
Skin diseases	161	8,53**	2.26	1.43–3.56	< .001	2.12	1.30–3.47	.003
Musculosceletal diseases	5661	8,33**	1.99	1.61–2.45	< .001	1.93	1.53–2.42	< .001
Genitourinary diseases	199	8,71**	2.64	1.76–3.96	< .001	2.40	1.54–3.76	< .001
Other diseases	442	8,34**	2.14	1.55–2.95	< .001	2.18	1.54–3.08	< .001

^1^ANOVA: Disease vs. reference means

* p < .01

** p < .001.

^2^ Adjusted with continuous BMI, 3-category smoking and alcohol use, 2-category physical activity and marital status.

## Results

### 3.1 Baseline characteristics

The mean LS score was 8.09 (SD 2.81) for the whole study population (N = 11,084). Those identified as dissatisfied had the highest morbidity and BMI. They were also physically less active, more likely to smoke and be non-drinkers than others (Table 1).

### 3.2 Morbidity

The overall risk (OR) for life dissatisfaction (LS 12–20 vs. LS 4–11) among the sick compared to the healthy was 1.77 (95%CI 1.44–2.18). The mean LS score increased along with number of diseases. For groups with 1–2 diseases, 3–4 diseases and ≥5 diseases the mean LS scores were 7.83, 8.37 and 9.07, respectively (each p < .001).

The risk of belonging to the intermediate (LS 7–11 vs. LS 4–6) or to the dissatisfied group (LS 12–20 vs. LS 4–6) according to number of diseases was examined with those with *no diseases* as a reference group (Table 2). Increasing number of diseases was strongly associated with life dissatisfaction. In each of the comparisons, both before and after adjusting for lifestyle factors (continuous BMI, 3-category smoking and alcohol use and 2-category physical activity and marital status), the differences were statistically significant. Patients with 5 or more chronic medical disorders had 4.53 (95%CI 3.31–6.21) times higher risk of life dissatisfaction compared with the healthy. When considering number of diseases as a continuous variable, risk of life dissatisfaction increased by 19.1% for each additional disease (OR_adj_ = 1.19; 95%CI 1.15–1.23). Adjustments did not considerably affect the risk estimates.

### 3.3 Disease main classes and specific disorders

The risk of belonging to the dissatisfied group [2-category LS (LS 12–20 vs. LS 4–11)] was investigated according to ICD main classes (Table 3) and by specific diseases. The crude ORs each of the ICD main classes were significant and the patterns of associations were sustained after applying full adjustments.

The highest risk for life dissatisfaction (OR_adj_ = 4.26; 95%CI 2.94–6.15) was found for patients with *mental and behavioral disorders* (main disease class). Patients with *alcoholism* (specific disease, n = 24) (OR_adj_ = 6.50; 95%CI 2.39–17.70) and self-reported *psychological symptoms* (i.e. insomnia and mood related symptoms, n = 35) (OR_adj_ = 4.32; 95%CI 1.88–9.94) scored the highest odds ratios.

Women with *neurological disorders* (main disease class) had the second highest risk of life dissatisfaction with the OR_adj_ of 3.36 (95%CI 2.33–4.84). For patients with *Parkinson’s disease or multiple sclerosis* (specific diseases, n = 48) the combined OR_adj_ was 4.51 (95%CI 2.24–9.06). Women reporting *other pain* (reported symptoms included in the *Other diseases* category, n = 27) had an OR_adj_ of 5.58 (95%CI 2.38–13.10).

Third highest risk of life dissatisfaction was found for *eye diseases* (disease main class) with the OR_adj_ of 3.07 (95%CI 2.11–4.48). Women with *visual impairment* (specific disease, n = 155) scored an OR_adj_ of 3.74 (95%CI 2.35–5.98). They were also 48.8% (p < .001) more likely to suffer from diabetes and/or hypertension than women who are not visual impaired.

### 3.4 Morbidity adjustments and single disease analyses

When adjusting for number of diseases, only patients with *mental and behavioral disorders* (OR_adj_ = 3.90, p < .001), *neurological disorders* (OR_adj_ = 1.95, p = .036) and *genitourinary diseases* (OR_adj_ = 2.54, p = .027) had a significantly increased risk of belonging to the dissatisfied group.

When excluding subjects having diseases from several ICD main classes, only patients with *mental and behavioral disorders* (OR_adj_ = 3.39, p = .004), *musculoskeletal diseases* (OR_adj_ = 1.49, p = .003) and *digestive system disorders* (OR_adj_ = 2.46, p = .040) had a significantly increased risk of belonging to the dissatisfied group.

## Discussion

The main finding to emerge from this population-based study was that multimorbidity has a strong dose-response association with life dissatisfaction among postmenopausal women. Women with five or more diseases had approximately five times greater risk of dissatisfaction than the healthy. In addition, those women with mental, neurological or eye diseases were identified as most dissatisfied, but each of the twelve ICD main disease classes were significantly associated with life dissatisfaction. Adjustment for multimorbidity altered somewhat this pattern, but not for *mental and behavioral disorders*, *neurological disorders and genitourinary diseases*.

Literature on the relationship between morbidity and SBW in general suggests that morbidity is associated with life dissatisfaction [10–13,36], adversely affects health-related quality of life [37] and correlates with depressive symptoms [38]. Somatic multimorbidity has been associated with a higher risk of mental disorders [23]. The present study is in accordance with previous studies suggesting that psychiatric disorders and symptoms are related with the most severe life dissatisfaction [10,11,25,39]. It was seen in respect to main disease classes (*mental and behavioral disorders*) and to specific diseases (*alcoholism*), however the present study also suggests that multimorbidity might be a more important factor with respect to life satisfaction than the type of disease itself.

Somatic symptoms are also related to life satisfaction and psychological symptoms. Terauchi et al. found that headache was associated with depression and nausea and that numbness associated with anxiety [40]. Broe et al. found that disorders which cause disability, such as Parkinson’s disease, heart disease and chronic lung disease, affect life satisfaction strongly [41]. Neurological disorders, such as Parkinson’s disease and multiple sclerosis, may cause physical disability, but also psychological symptoms such as depression [42]. Previously, metabolic syndrome has been associated with depressive symptoms, at least in men [43]. In the present study among women, *overweight* (as a reported disease) as well as high BMI were associated with life dissatisfaction. The latter association vanished in some of the fully adjusted models, but not in all.

In the present study, some diseases and disease groups–i.e. *mental and behavioral disorders*, *neurological disorders* and *eye diseases*—were more strongly associated with life dissatisfaction than the others. Even though some health disorders and symptoms has been studied by life satisfaction (11, 12), only one previous report (data derived from a Finnish dissertation) has compared life satisfaction systematically by ICD main classes [39]. This study utilized retrospective hospital discharge data within three years (1972–1975) before life satisfaction assessments with the 4-item LS-scale for the Finnish Twin cohort consisting of men and women aged 18–64 years [39]. Patients with mental disorders had the highest life dissatisfaction, followed by patients with neoplasms. Eye diseases were not specified. In the present study, *neoplasms* were not as strongly associated with life dissatisfaction, but the aging women reported a lifetime history of diseases, which included mostly less severe forms of cancer i.e. those which have allowed them to stay alive. In general, not only disease severity, but also availability of efficient treatment choices can affect the psychological burden of the diseases over time. Still, when comparing two studies within 25 year time lap, it seems to verify that mental problems jeopardize most heavily life satisfaction.

The present study showed that women with an eye disease were dissatisfied. A main cause of eye problems at this age is retinopathy due to hypertension or type II diabetes [44,45]. These diseases as a rule are not strongly related to dissatisfaction, only the most severe cases. On the other hand, multimorbidity plays a role in this, since our subjects reporting *visual impairment* had significantly (p < .001) more often diabetes and/or hypertension than the rest of the study population.

The strengths of the present study include a large population-based cohort, high response rate and the opportunity to adjust for several potential confounding factors. The narrow age range increased the homogeneity of the sample and provides important information on aging women. Overall response rate was high (81.1%) and only 3.9% of participants did not respond to the LS questions. However, the cross-sectional design of the present study did not allow the investigation of the causal relationship between morbidity and life satisfaction, which is likely to be bidirectional [46].

The number of subjects with medical conditions from only one disease class was relatively small, which might result in type II error. There might also be potential bias in self-reports of health disorders due to incomplete recall or understanding of health disorders. The women were 57–66 years old at the time of enquiry. Memory problems at this age are not common, but symptomless diseases and diseases not requiring medication may be underreported. Still, self-reported medical conditions diagnosed by a physician have good concordance with medical records [47]. Further, postal inquiry seems to be a more relevant way to investigate SWB than interviews which are more prone to response bias caused by e.g. incorrect interpretation of the interviewer or response bias caused by self-presentation interviewee [48].

Retirement is an important milestone in older adulthood, and there is no single way how it affects life satisfaction [49]. The present study discusses life satisfaction among postmenopausal women within the age range of 57–66 years. Altogether 74.2% of study population reported themselves as being retired. However, within this age range, we found no significant difference in mean LS between retired and working persons. On the other hand, marital status was strongly associated with LS, but did not, however, affect the relationship between morbidity and LS.

## Conclusions

In conclusion, a strong disease-specific and dose-dependent relationship was observed between morbidity and life dissatisfaction. Interestingly, disease burden seemed to be a more important factor relating to life satisfaction than type of disease. Patients with mental and neurological disorders had the highest risk of life dissatisfaction. Even though tentative conclusions could be drawn, further investigations determining causality of the observed associations are warranted. Still, monitoring life satisfaction among aging women, regardless of disorder, should be undertaken in order to intervene the adverse, joint effects, of poor health and poor well-being.

## Supporting Information

S1 Dataset (XLSX)

## Abbreviations

LS: life satisfaction
SWB: subjective well-being
BMI: body mass index
OR: odds ratio
